# Use of Health Information Technology by Adults With Diabetes in the United States: Cross-sectional Analysis of National Health Interview Survey Data (2016-2018)

**DOI:** 10.2196/27220

**Published:** 2022-01-12

**Authors:** Seamus Y Wang, Hsin-Chieh Yeh, Arielle Apfel Stein, Edgar R Miller

**Affiliations:** 1 College of Arts and Sciences University of Pennsylvania Philadelphia, PA United States; 2 Department of Medicine Johns Hopkins University Baltimore, MD United States

**Keywords:** health information technology, National Health Interview Survey, diabetes, Healthy People 2020, Healthy People 2030, mobile phone

## Abstract

**Background:**

The use of health information technology (HIT) has been proposed to improve disease management in patients with type 2 diabetes mellitus.

**Objective:**

This study aims to report the prevalence of HIT use in adults with diabetes in the United States and examine the factors associated with HIT use.

**Methods:**

We analyzed data from 7999 adults who self-reported a diabetes diagnosis as collected by the National Health Interview Survey (2016-2018). All analyses were weighted to account for the complex survey design.

**Results:**

Overall, 41.2% of adults with diabetes reported looking up health information on the web, and 22.8% used eHealth services (defined as filled a prescription on the web, scheduled an appointment with a health care provider on the web, or communicated with a health care provider via email). In multivariable models, patients who were female (vs male: prevalence ratio [PR] 1.16, 95% CI 1.10-1.24), had higher education (above college vs less than high school: PR 3.61, 95% CI 3.01-4.33), had higher income (high income vs poor: PR 1.40, 95% CI 1.23-1.59), or had obesity (vs normal weight: PR 1.11, 95% CI 1.01-1.22) were more likely to search for health information on the web. Similar associations were observed among age, race and ethnicity, education, income, and the use of eHealth services. Patients on insulin were more likely to use eHealth services (on insulin vs no medication: PR 1.21, 95% CI 1.04-1.41).

**Conclusions:**

Among adults with diabetes, HIT use was lower in those who were older, were members of racial minority groups, had less formal education, or had lower household income. Health education interventions promoted through HIT should account for sociodemographic factors.

## Introduction

### Background

Advances in technologies have introduced mechanisms that support effective and affordable health care delivery and education. In recent years, industries and health care systems have made significant efforts to expand health technology for people with diabetes. Mobile apps and web-based platforms provide many options for managing diabetes, including blood glucose tracking, insulin dosing, and diabetes education [1]. Web-based patient portals improve access to health information and personal health records. Although these tools have shown promise, particularly by improving glycemic control and reducing hemoglobin A_1c_ levels, the effectiveness of these interventions at the population level is reliant on actual use by people with diabetes [2,3].

The Department of Health and Human Services has established the Healthy People initiatives to promote public health and well-being priorities across the United States by providing measurable, decade-long public health objectives [4]. Healthy People 2020 specified the objectives of using health information technology (HIT) to improve population health outcomes and health care quality and to achieve health equity [1]. These specific objectives included increasing the use of electronic personal health management tools (HIT Objective 5.1), increasing the use of the internet to communicate with their health providers (HIT Objective 5.2), and increasing web-based health information seeking (HIT Objective 9). In addition, the published Healthy People 2030 goals have since been built upon the established 2020 HIT goals. The new 2030 goals underscore a desire to increase the use of patient portals, particularly the proportion of adults who use information technology to track health care data or communicate with health care providers [5].

A study using data from the Health Information National Trends Survey (HINTS; 2014-2017) reported that 80% of survey participants went on the web to access the internet or to send and receive email, more than 70% had broadband access, and more than 65% had access via cellular network [6]. However, these statistics were not specific to health-related information seeking or communication. Little is known about the proportion of people with diabetes who use the internet to search for health information on the web and communicate with health care providers. In addition, less attention has been paid to how HIT use in people with type 2 diabetes compares to US National Public Health objectives. There is a need for this information, as people with diabetes often have complex management needs and potentially face barriers to HIT but may benefit greatly from eHealth services.

In addition, reporting the association between sociodemographic factors and HIT use by people with diabetes may provide new insight to better promote this technological approach to care. It is well-known that patients of older age, lower socioeconomic status, lower level of education, and racial or ethnic minorities are less likely to engage in eHealth activities, such as looking up health information on the web [1,7,8]. Patients with type 2 diabetes tend to be even more disadvantaged than those in the general population [9,10]. It is therefore important to understand the sociodemographic factors that influence HIT use in patients with diabetes to assist vulnerable populations and advance the progress of health equity.

### Objective

The aim of this study is to examine the prevalence of HIT use in adults with diabetes in the United States, compare it with the goals set in Healthy People 2020, and identify factors associated with HIT use by analyzing data from the National Health Interview Survey (NHIS; 2016-2018), which provides a large, nationally representative sample.

## Methods

### Study Design and Setting

The NHIS provides data on the health status, health care access, and health behavior of the noninstitutionalized civilian population in the United States using a multistage probability sampling design. The data were collected by trained interviewers using a computer-assisted personal interviewing program and were based on self-reports from the respondents. Details regarding the study design, questionnaires, and procedures are available elsewhere [11].

We used data collected between 2016 and 2018 from a sample of NHIS adult participants who self-reported diabetes diagnosed after an age of 25 years and had a BMI ≥18.5 kg/m^2^. As types of diabetes were not asked consistently in all survey years during 2016-2018, we were unable to completely distinguish between responders with type 1 and type 2 diabetes. However, because 90%-95% of all adults with diabetes are type 2, and type 1 is more commonly diagnosed at earlier ages, we are confident that the study participants in our sample most likely had type 2 diabetes [10,12].

Survey participants were asked whether they had ever used a computer in the last 12 months for any of the following tasks: (1) to look up health information on the internet, (2) to fill a prescription, (3) to schedule an appointment with a health care provider on the web, or (4) to communicate with a health care provider via email. We studied 2 primary outcomes: prevalence of participants who ever looked up health information on the web and prevalence of participants who ever used eHealth services. We categorized a patient as ever using eHealth services if the individual reported ever scheduling appointments, communicating with health care providers, or refilling prescription medications on the web [13]. The prevalence of using each of the 3 components of eHealth services was also examined.

In a separate question, survey participants were asked, “Do you use the Internet?” We conducted a subgroup analysis on HIT use only by adults with diabetes who indicated that they were internet users.

Sociodemographic variables of interest were age, sex, race and ethnicity, educational attainment, health insurance coverage status, and income to poverty ratio. Individuals were classified into 3 categories according to calculated BMI: normal weight (18.5 kg/m^2^≥ BMI <25 kg/m^2^), overweight (25 kg/m^2^ BMI <30 kg/m^2^), or obese (BMI 30 kg/m^2^). BMI was calculated based on self-reported height and weight. The use of antidiabetic medications was assessed based on self-reports and was classified as no medication use, oral medication only, or any insulin use. Other self-report variables included history of chronic disease and having at least one visit to a doctor or health care professional in the past year.

### Statistical Analysis

We pooled 3 years of data from 2016 to 2018 and created new design variables incorporating stratum, primary sampling unit, and sampling weight. This approach accounted for complex sampling designs and weights and was limited to eligible adults with diabetes using the STATA (StataCorp LP) *subpop* command for correct SE estimation. Baseline characteristics according to HIT use were compared using chi-square tests for categorical variables (eg, age group, sex, race and ethnicity, educational attainment, and income). Multiple Poisson regression models were constructed to estimate prevalence ratios (PRs) and their 95% CIs and examine the association between BMI category, sociodemographic characteristics, and HIT use in adults with diabetes, adjusting for covariates.

All analyses were weighted to account for the complex survey design. All tests of significance were 2-tailed with an α level of .05. Analyses were performed using Stata 14 (StataCorp LLC).

## Results

### Overview

We identified 7999 individuals who reported diabetes diagnosed after the age of 25 years with BMI ≥18.5 kg/m^2^. Overall, 41.2% of adults with diabetes looked up health information on the web, and 22.8% used eHealth services (14.7% filling a prescription on the web, 12.2% scheduling an appointment on the web, and 15% communicating with a health care provider via email).

Non-Hispanic White and female adults with diabetes were more likely to search for health information on the web. Graded relationships were used to search for health information on the web across categories of age, education level, and income. Adults with obesity and those not on any medications were more likely to look up health information on the web.

For eHealth services use, higher proportions of non-Hispanic White and Asian populations used eHealth services than other racial and ethnic groups. Graded relationships also existed for using eHealth services across age groups, education levels, and income levels. There were no differences by sex, BMI, or antidiabetic medication status.

Compared with individuals who did not look up health information on the web, adults with diabetes who looked up health information on the web were more likely to be <65 years of age, female, and non-Hispanic White. They were more likely to be of higher education and higher income, more likely to have obesity and see or talk to health care providers in the past 12 months, and less likely to be on antidiabetic medications. They were also less likely to have cardiovascular disease and cancer (Table 1). The 2 groups did not differ by insurance status or arthritis status.

**Table 1:** Participant characteristics by health information technology use (National Health Interview Survey, 2016-2018; N=7999).

Characteristics		Look up web-based health information, % (SE)		*P* value		Use eHealth services, % (SE)				*P* value	
		No (n=4706)	Yes (n=3293)			No (n=6174)		Yes (n=1825)			
**Age group (years)**					<.001						<.001
	25-44	8.22 (0.6)	13.45 (0.8)			9.61 (0.56)		13.11 (1.03)			
	45-64	42.13 (0.95)	53.67 (1.05)			45.2 (0.82)		52.86 (1.4)			
	≥65	49.65 (0.92)	32.88 (0.94)			45.2 (0.79)		34.03 (1.31)			
**Sex**					<.001						.12
	Male	54.71 (0.94)	49.61 (1.09)			51.87 (0.82)		54.56 (1.42)			
	Female	45.29 (0.94)	50.39 (1.09)			48.13 (0.82)		45.44 (1.42)			
**Race and ethnicity**					<.001						<.001
	Non-Hispanic White	52.53 (1.43)	69.52 (1.14)			55.21 (1.33)		74.01 (1.47)			
	Non-Hispanic Black	16.55 (0.94)	12.17 (0.78)			16.48 (0.86)		9.08 (0.92)			
	Non-Hispanic Asian	5.47 (0.54)	4.88 (0.55)			4.77 (0.45)		6.59 (0.9)			
	Non-Hispanic others	3.54 (0.68)	2.87 (0.35)			3.68 (0.61)		1.93 (0.35)			
	Hispanic	21.92 (1.33)	10.57 (0.87)			19.87 (1.17)		8.4 (0.96)			
**Education level**					<.001						<.001
	Less than high school	27.85 (0.94)	6.9 (0.66)			23.56 (0.81)		4.38 (0.59)			
	High school	33.13 (0.95)	21.49 (0.93)			31.18 (0.78)		18.74 (1.21)			
	Some college	25.43 (0.83)	37.22 (1.11)			28.64 (0.8)		36.18 (1.45)			
	College	8.53 (0.54)	19.77 (0.91)			10.1 (0.49)		23.34 (1.23)			
	Above college	4.18 (0.34)	14.29 (0.76)			5.78 (0.36)		16.97 (1.09)			
	Do not know	0.89 (0.21)	0.34 (0.17)			0.74 (0.17)		0.39 (0.23)			
**Income to poverty ratio**					<.001						<.001
	Poor (<100% FPL^a^)	17.17 (0.80)	8.64 (0.62)			15.95 (0.67)		5.99 (0.71)			
	Near poor (100%-199% FPL)	25.25 (0.84)	14.59 (0.76)			24.04 (0.75)		10.31 (0.85)			
	Middle income (200%-399% FPL)	26.94 (0.84)	27.92 (1.01)			27.54 (0.75)		26.81 (1.28)			
	High income (≥400% FPL)	23.2 (0.89)	42.5 (1.22)			24.99 (0.79)		51.5 (1.51)			
	Do not know	7.45 (0.55)	6.34 (0.57)			7.49 (0.48)		5.39 (0.74)			
**BMI category**					<.001						.005
	Normal	12.81 (0.62)	10.46 (0.67)			12.03 (0.53)		11.1 (0.95)			
	Overweight	31.93 (0.93)	26.77 (0.96)			30.8 (0.81)		26.37 (1.3)			
	Obese	55.26 (0.98)	62.77 (1.04)			57.17 (0.84)		62.53 (1.37)			
**Insurance**					.48						.002
	Insured	93.42 (0.57)	93.97 (0.61)			92.88 (0.51)		96.04 (0.73)			
	Uninsured	6.58 (0.57)	6.03 (0.61)			7.12 (0.51)		3.96 (0.73)			
**Hypertension**					<.001						.02
	No	24.81 (0.82)	29.65 (1.08)			26 (0.69)		29.62 (1.43)			
	Yes	75.19 (0.82)	70.35 (1.08)			74 (0.69)		70.38 (1.43)			
**CHD^b^**					<.001						<.001
	No	78.52 (0.72)	83.89 (0.77)			79.59 (0.63)		84.62 (1.05)			
	Yes	21.48 (0.72)	16.11 (0.77)			20.41 (0.63)		15.38 (1.05)			
**Stroke**					<.001						<.001
	No	89.04 (0.61)	93.03 (0.51)			89.74 (0.52)		93.89 (0.62)			
	Yes	10.96 (0.61)	6.97 (0.51)			10.26 (0.52)		6.11 (0.62)			
**Arthritis**					.13						.72
	No	52.75 (0.96)	50.68 (1.01)			52.01 (0.84)		51.4 (1.45)			
	Yes	47.25 (0.96)	49.32 (1.01)			47.99 (0.84)		48.6 (1.45)			
**Cancer**					.045						.02
	No	84.48 (0.63)	82.54 (0.77)			84.28 (0.54)		81.7 (1.05)			
	Yes	15.52 (0.63)	17.46 (0.77)			15.72 (0.54)		18.3 (1.05)			
**Seen or talked to a general physician or specialist in the past 12 months**					<.001						<.001
	No	13.54 (0.67)	9.76 (0.63)			13.28 (0.59)		7.7 (0.74)			
	Yes	86.46 (0.67)	90.25 (0.63)			87.72 (0.59)		92.3 (0.74)			
**Antidiabetic medication status**					.002						.62
	No medication	13.37 (0.67)	16.92 (0.89)			15.17 (0.66)		14.02 (1.03)			
	Oral medication only	57.35 (0.97)	55.88 (1.08)			56.48 (0.84)		57.45 (1.4)			
	Insulin treatment	29.29 (0.88)	27.2 (0.94)			28.35 (0.73)		28.53 (1.26)			

^a^FPL: federal poverty level.

^b^CHD: coronary heart disease (includes coronary heart disease, angina, or heart attack).

Similar associations were seen when comparing adults with diabetes who used eHealth services to those who did not use eHealth services. However, those who used eHealth services were more likely to have insurance than those who did not use eHealth services. There were no significant differences between the users and nonusers of eHealth services by sex or antidiabetic medication use.

In the multivariable model that included age group, sex, race and ethnicity, education level, income to poverty ratio category, BMI category, prevalent chronic conditions, provider visit, insurance, and antidiabetic medication use, patients who were female (vs male: PR 1.16, 95% CI 1.10-1.24), had higher education (above college vs less than high school: PR 3.61, 95% CI 3.01-4.33), had higher income (high income vs poor: PR 1.40, 95% CI 1.23-1.59), or were obese (vs normal weight: PR 1.11, 95% CI 1.01-1.22), were more likely to search for health information on the web. Adults with diabetes who were >45 years or racial minorities were less likely to search for health information on the web (Table 2). Similar associations were observed between sociodemographic characteristics and the use of eHealth services. In contrast to the univariate analysis, in the multivariable model, patients on insulin were more likely to use eHealth services (on insulin vs no medication: PR 1.21, 95% CI 1.04-1.41) after considering other covariates. There were no significant differences between men and women and across BMI categories regarding the use of eHealth services (Table 2).

**Table 2:** Proportions and adjusted prevalence ratios (PRs; 95% CI) of health information technology use by sociodemographic characteristics, BMI category, and medication status (National Health Interview Survey, 2016-2018).

Characteristics			Look up web-based health information^a^					Use eHealth services^a^		
			Unadjusted % (SE)		Adjusted PR (95% CI)		Unadjusted % (SE)			Adjusted PR (95% CI)
**Age group (years)**										
	25-44	55.06 (2.37)		1.00 (reference)		30.62 (2.16)			1.00 (reference)	
	45-64	48.83 (1.06)		0.82 (0.75-0.90)^b^		27.44 (1.02)			0.76 (0.66-0.88)^b^	
	≥65	33.16 (0.98)		0.55 (0.50-0.61)^b^		19.58 (0.83)			0.52 (0.44-0.61)^b^	
**Sex**										
	Male	40.45 (1.02)		1.00 (reference)		25.38 (1)			1.00 (reference)	
	Female	45.46 (1.08)		1.16 (1.10-1.24)^b^		23.39 (0.88)			1.01 (0.92-1.11)	
**Race and ethnicity**										
	Non-Hispanic White	49.79 (0.9)		1.00 (reference)		30.24 (0.94)			1.00 (reference)	
	Non-Hispanic Black	35.52 (1.79)		0.79 (0.72-0.87)^b^		15.12 (1.43)			0.61 (0.50-0.73)^b^	
	Non-Hispanic Asian	40.07 (3.28)		0.80 (0.68-0.94)^b^		30.9 (3.57)			0.99 (0.80-1.22)	
	Non-Hispanic others	37.79 (4.38)		0.83 (0.70-0.98)^b^		14.47 (3.1)			0.61 (0.43-0.86)^b^	
	Hispanic	26.54 (1.88)		0.71 (0.62-0.81)^b^		12.02 (1.4)			0.61 (0.49-0.77)^b^	
**Education level**										
	Less than high school	15.65 (1.41)		1.00 (reference)		5.67 (0.76)			1.00 (reference)	
	High school	32.7 (1.31)		1.79 (1.48-2.17)^b^		16.27 (1.04)			2.16 (1.61-2.88)^b^	
	Some college	52.29 (1.31)		2.64 (2.21-3.16)^b^		29.01 (1.27)			3.37 (2.57-4.41)^b^	
	College	63.5 (1.93)		3.20 (2.65-3.85)^b^		42.78 (1.97)			4.55 (3.43-6.03)^b^	
	Above college	71.9 (1.97)		3.61 (3.01-4.33)^b^		48.72 (2.34)			4.95 (3.74-6.54)^b^	
**Income to poverty ratio**										
	Poor (<100% FPL^c^)	27.39 (1.83)		1.00 (reference)		10.84 (1.29)			1.00 (reference)	
	Near poor (100%-199% FPL)	30.21 (1.43)		1.06 (0.92-1.22)		12.18 (1.05)			1.03 (0.79-1.36)	
	Middle income (200%-399% FPL)	43.7 (1.38)		1.31 (1.15-1.50)^b^		23.94 (1.18)			1.62 (1.28-2.03)^b^	
	High income (≥400% FPL)	57.85 (1.34)		1.40 (1.23-1.59)^b^		40 (1.32)			2.02 (1.60-2.54)^b^	
**BMI category**										
	Normal	37.95 (1.9)		1.00 (reference)		22.99 (1.8)			1.00 (reference)	
	Overweight	38.58 (1.27)		1.03 (0.93-1.14)		21.69 (1.17)			0.97 (0.82-1.14)	
	Obese	45.97 (1)		1.11 (1.01-1.22)^b^		26.13 (0.87)			1.10 (0.95-1.26)	
**Antidiabetic medication status**										
	No medication	48.67 (1.92)		1.00 (reference)		23.02 (1.67)			1.00 (reference)	
	Oral medication	42.2 (0.97)		0.95 (0.88-1.03)		24.75 (0.88)			1.14 (0.98-1.31)	
	Insulin treatment	41.03 (1.32)		0.97 (0.89-1.06)		24.55 (1.13)			1.21 (1.04-1.41)^b^	

^a^Models include BMI category, age group, sex, race and ethnicity, education level, income to poverty ratio category, prevalent chronic conditions, health care provider visit, and insurance.

^b^Statistically significant based on a 95% CI.

^c^FPL: federal poverty level.

### Subgroup Analysis of Internet Users Only

We conducted a subgroup analysis of the 4805 adults (weighted percentage 62.3%, SE 0.83%, of all adults with diabetes) who reported using the internet.

Among internet users, 64.6% (SE 0.87%) reported looking up health information and 37.3% (SE 0.95%) reported using eHealth services, including 22.4% (SE 0.76%) who filled a prescription on the web, 18.5% (SE 0.75%) who scheduled a medical appointment on the web, and 23.2% (SE 0.85%) who communicated with a health care provider via email.

Table 3 shows associations between HIT use and individual characteristics among internet users with diabetes. Compared with internet users who did not search for health information on the web, users who searched for health information on the web were more likely to be <65 years old, female, non-Hispanic White, of higher education level and higher income, have obesity, and more likely to see or talk to providers. There were no associations with chronic conditions other than arthritis and antidiabetic medication use.

**Table 3:** Participant characteristics by health information technology use among internet users (National Health Interview Survey, 2016-2018; N=7999).

Characteristics		Look up web-based health information, % (SE)			*P* value			Use eHealth services, % (SE)					*P* value	
		No (n=1720)	Yes (n=3085)				No (n=3071)			Yes (n=1734)				
**Age group (years)**						.004								.55
	25-44	13.94 (1.17)	13.14 (0.8)				13.75 (0.91)			12.86 (1.03)				
	45-64	48.08 (1.59)	54.21 (1.09)				51.31 (1.17)			53.28 (1.44)				
	≥65	37.98 (1.44)	32.65 (0.96)				34.93 (1.01)			33.86 (1.35)				
**Sex**						<.001								.13
	Male	58.63 (1.39)	49.76 (1.1)				51.82 (1.11)			54.71 (1.44)				
	Female	41.37 (1.39)	50.24 (1.1)				48.18 (1.11)			45.29 (1.44)				
**Race and ethnicity**						<.001								<.001
	Non-Hispanic White	62.22 (1.72)	70.05 (1.18)				63.2 (1.39)			74.15 (1.5)				
	Non-Hispanic Black	12 (1.05)	11.66 (0.8)				13.36 (0.89)			9.12 (0.96)				
	Non-Hispanic Asian	6.43 (0.92)	5.12 (0.58)				4.92 (0.6)			6.69 (0.92)				
	Non-Hispanic others	2.96 (0.56)	2.92 (0.37)				3.5 (0.5)			1.99 (0.37)				
	Hispanic	16.39 (1.34)	10.25 (0.88)				15.02 (1.09)			8.06 (0.95)				
**Education level**						<.001								<.001
	Less than high school	11.55 (0.99)	6.32 (0.63)				10.81 (0.79)			3.73 (0.56)				
	High school	29.25 (1.35)	21.01 (0.97)				27.13 (0.97)			18.53 (1.25)				
	Some college	36.14 (1.4)	37.5 (1.15)				37.58 (1.15)			36.06 (1.51)				
	College	14.92 (1.09)	20.14 (0.94)				14.98 (0.82)			23.87 (1.26)				
	Above college	7.8 (0.76)	14.68 (0.78)				9.18 (0.62)			17.41 (1.12)				
	Do not know	0.32 (0.14)	0.36 (0.18)				0.31 (0.15)			0.41 (0.24)				
**Income to poverty ratio**						.002								<.001
	Poor (<100% FPL^a^)	8.11 (0.78)	8.66 (0.65)				10.05 (0.7)			5.79 (0.73)				
	Near poor (100%-199% FPL)	18.65 (1.12)	13.7 (0.76)				18.8 (0.9)			9.83 (0.83)				
	Middle income (200%-399% FPL)	28.2 (1.3)	27.96 (1.04)				28.95 (1.04)			26.52 (1.32)				
	High income (≥400% FPL)	38.09 (1.55)	43.39 (1.24)				35.05 (1.18)			52.38 (1.55)				
	Do not know	6.95 (0.79)	6.29 (0.59)				7.14 (0.61)			5.48 (0.77)				
**BMI category**						.006								.16
	Normal	10.6 (0.92)	10.41 (0.68)				10.21 (0.7)			10.93 (0.97)				
	Overweight	31.75 (1.54)	26.67 (1)				29.7 (1.16)			26.39 (1.35)				
	Obese	57.65 (1.63)	62.92 (1.07)				60.09 (1.2)			62.68 (1.43)				
**Insurance**						.66								.003
	Insured	93.53 (0.92)	94 (0.63)				92.57 (0.7)			95.96 (0.77)				
	Uninsured	6.47 (0.92)	6 (0.63)				7.43 (0.7)			4.04 (0.77)				
**Hypertension**						.33								.61
	No	31.25 (1.39)	29.55 (1.14)				30.48 (1.07)			29.6 (1.46)				
	Yes	68.75 (1.39)	70.45 (1.14)				69.52 (1.07)			70.4 (1.46)				
**CHD^b^**						.14								.17
	No	82.43 (1.06)	84.33 (0.76)				82.93 (0.81)			84.88 (1.08)				
	Yes	17.57 (1.06)	15.67 (0.76)				17.07 (0.81)			15.12 (1.08)				
**Stroke**						.86								.08
	No	93.08 (0.74)	93.24 (0.52)				92.59 (0.58)			94.17 (0.62)				
	Yes	6.92 (0.74)	6.76 (0.52)				7.41 (0.58)			5.83 (0.62)				
**Arthritis**						<.001								.06
	No	58.87 (1.42)	50.79 (1.04)				54.97 (1.09)			51.43 (1.49)				
	Yes	41.13 (1.42)	49.21 (1.04)				45.03 (1.09)			48.57 (1.49)				
**Cancer**						.17								.06
	No	84.42 (1.09)	82.56 (0.79)				84.13 (0.77)			81.69 (1.08)				
	Yes	15.58 (1.09)	17.44 (0.79)				15.87 (0.77)			18.31 (1.08)				
**Seen or talked to a general physician or specialist in the past 12 months**						.004								<.001
	No	13.01 (1.03)	9.73 (0.66)				12.82 (0.8)			7.64 (0.77)				
	Yes	86.99 (1.03)	90.27 (0.66)				87.18 (0.8)			92.36 (0.77)				
**Antidiabetic medication status**						.17								.02
	No medication	15.09 (1.13)	17.03 (0.93)				17.7 (1)			14.06 (1.07)				
	Oral medication	59.57 (1.49)	56.24 (1.09)				57.25 (1.2)			57.69 (1.44)				
	Insulin treatment	25.34 (1.33)	26.73 (0.95)				25.05 (0.99)			28.25 (1.29)				

^a^FPL: federal poverty level.

^b^CHD: coronary heart disease.

Compared with users who did not use eHealth services, users who used eHealth services were more likely to be non-Hispanic White, had higher education and higher income, were more likely to have insurance, were more likely to be taking antidiabetic medication, and were more likely to see a physician in the past 12 months. There were no associations between eHealth service use and age, sex, BMI category, or comorbidities.

In the multivariable model, being female or having higher education was associated with being more likely to search for health information on the web, whereas being ≥65 years, Hispanic, or near poor was associated with being less likely to search for health information on the web (Table 4).

**Table 4:** Proportions and prevalence ratios (PRs) of health information technology use by sociodemographic characteristics, obesity category, and antidiabetic medication status in internet users (National Health Interview Survey, 2016-2018).

Characteristics		Look up web-based health information^a^			Use eHealth services^a^	
		Unadjusted % (SE)	Adjusted PR (95% CI)	Unadjusted % (SE)		Adjusted PR (95% CI)
**Age group (years)**						
	25-44	63.27 (2.49)	1.00 (reference)	35.74 (2.56)		1.00 (reference)
	45-64	67.34 (1.21)	1.00 (0.92-1.09)	38.19 (1.34)		0.88 (0.76-1.02)
	≥65	61.11 (1.38)	0.87 (0.79-0.95)^b^	36.58 (1.34)		0.75 (0.64-0.88)^b^
**Sex**						
	Male	60.81 (1.21)	1.00 (reference)	38.58 (1.36)		1.00 (reference)
	Female	68.94 (1.23)	1.14 (1.08-1.20)^b^	35.87 (1.25)		0.99 (0.90-1.08)
**Race and ethnicity**						
	Non-Hispanic White	67.3 (0.96)	1.00 (reference)	41.11 (1.17)		1.00 (reference)
	Non-Hispanic Black	63.97 (2.55)	0.93 (0.86-1.01)	28.87 (2.48)		0.73 (0.62-0.87)^b^
	Non-Hispanic Asian	59.27 (4.3)	0.87 (0.75-1.01)	44.73 (4.67)		1.02 (0.83-1.25)
	Non-Hispanic others	64.3 (4.8)	0.94 (0.82-1.07)	25.26 (4.45)		0.67 (0.49-0.93)^b^
	Hispanic	53.35 (3.01)	0.85 (0.76-0.95)^b^	24.19 (2.67)		0.71 (0.57-0.88)^b^
**Education level**						
	Less than high school	50 (3.39)	1.00 (reference)	17.02 (2.41)		1.00 (reference)
	High school	56.76 (1.85)	1.13 (0.98-1.31)	28.9 (1.74)		1.49 (1.10-2.01)^b^
	Some college	65.47 (1.4)	1.29 (1.12-1.47)^b^	36.34 (1.53)		1.78 (1.34-2.37)^b^
	College	71.17 (1.97)	1.43 (1.25-1.65)^b^	48.66 (2.16)		2.26 (1.68-3.03)^b^
	Above college	77.47 (1.98)	1.58 (1.37-1.83)^b^	53.02 (2.48)		2.41 (1.80-3.23)^b^
**Income to poverty ratio**						
	Poor (<100% FPL^c^)	66.12 (2.74)	1.00 (reference)	25.51 (2.83)		1.00 (reference)
	Near poor (100%-199% FPL)	57.32 (2.18)	0.90 (0.81-0.99)^b^	23.73 (1.98)		0.92 (0.71-1.20)
	Middle income (200%-399% FPL)	64.44 (1.58)	0.98 (0.89-1.08)	35.27 (1.62)		1.26 (1.01-1.57)^b^
	High income (≥400% FPL)	67.56 (1.36)	0.97 (0.89-1.06)	47.06 (1.45)		1.47 (1.18-1.84)^b^
**BMI category**						
	Normal	64.23 (2.45)	1.00 (reference)	38.9 (2.76)		1.00 (reference)
	Overweight	60.57 (1.64)	0.96 (0.88-1.05)	34.59 (1.76)		0.92 (0.78-1.08)
	Obese	66.61 (1.12)	1.01 (0.93-1.10)	38.29 (1.14)		1.01 (0.88-1.16)
**Antidiabetic medication status**						
	No medication	67.35 (2.18)	1.00 (reference)	32.09 (2.31)		1.00 (reference)
	Oral medication	63.32 (1.12)	0.96 (0.89-1.03)	37.49 (1.2)		1.14 (0.99-1.32)
	Insulin treatment	65.64 (0.87)	1.00 (0.93-1.09)	40.15 (1.69)		1.26 (1.08-1.47)^b^

^a^Models include BMI category, age group, sex, race and ethnicity, education level, income to poverty ratio category, prevalent chronic conditions, health care provider visit, and insurance.

^b^Statistically significant based on a 95% CI.

^c^FPL: federal poverty level.

Similarly, having higher education or higher income was significantly associated with a higher likelihood of using eHealth services. Patients using insulin were more likely to use eHealth services. However, those who were ≥65 years, non-Hispanic Black, Hispanic, or other non-Hispanic races were less likely to use e-services. There were no associations between BMI category and searching for health information on the web or using eHealth services.

## Discussion

### Principal Findings

This study found that in adults with diabetes, those of younger age, higher income and education, and non-Hispanic White were more likely to search for information on the web and use eHealth services. Moreover, patients who had obesity were more likely to search for health information on the web and use eHealth services. Our study also found that patients on insulin were 21% more likely to use eHealth services compared with those not on medications.

Healthy People 2020 set a goal of 45% on the proportion of web-based health information seekers (HIT Objective 9) and a goal of 15% on the proportion of persons who use the internet to communicate with their health providers (HIT Objective 5.2) by the year 2020. Although Healthy People 2020 goals were set for the entire population and were not specific toward people with diabetes, our study indicated that adults with diabetes in the United States did not completely achieve these goals—41.2% of adults with diabetes were web-based health information seekers, and 15% of adults with diabetes used the internet to communicate with their health providers.

### Comparisons With Prior Surveys

Chou et al [13], using the NHIS (2009-2013) data, reported that in adults with diabetes, the multivariate-adjusted prevalence of scheduling appointments, communicating with health care providers, refilling prescriptions on the web, and any eHealth service use were 3.9%, 5.8%, 9%, and 13.8%, respectively. Compared with earlier data, our report from the NHIS demonstrates an overall increase in HIT use in adults with diabetes.

Our study found that only 62.3% of adults with diabetes reported using the internet in 2016-2018 (based on subgroup analyses). The proportion of internet users with diabetes is lower than the statistics reported in the general adult population, which increased from 87% in 2016 to 89% in 2018 [14]. In addition, a study using HINTS (2003-2017) data reported an increase from 14.2% to 70.9% between 2008 and 2017 in adults who reported tracking of electronic personal health information [15]. Together, these data suggest that individuals with diabetes have lower HIT use. Similarly, adults with diabetes tend to be older, racial minorities, and have lower education or lower income; these factors contribute to the lower accessibility and adaptability of internet use. Nonetheless, it is important to note that the survey response rate in the HINTS study was approximately 30%; we cannot rule out the possible effects of response bias on study outcomes. Our study used a data set with a higher response rate of approximately 70%.

Graetz et al [16] showed that ethnic minorities living in lower socioeconomic status neighborhoods were significantly more likely to access web-based personal health records through a mobile device, and as high as 19% relied exclusively on smartphones for internet access. Electronic health records (EHRs) and web-based interventions that are only available via a computer would not be available in mobile-exclusive populations, which may be more likely to include people with diabetes. This is another possible explanation for the lower use observed in people with diabetes.

### Closing the Digital Divide

Initiatives to assist internet access and awareness in older and underserved populations are needed to close the digital divide. Mobile access to web-based health services is increasingly being used by hospital systems and providers. SMS text messaging, a popular mobile media platform, is a possible candidate for health interventions. Mayberry et al [17] reported that interventions that use SMS text messaging services may be more accessible to people with diabetes when compared with internet-based platforms, as people with diabetes were found to use internet-dependent interventions less than those conducted through SMS text messages.

Consistent with earlier studies in adults with and without diabetes, lower HIT use was observed among adults who are older, racial minorities, less educated, and with lower income. A study in Norway analyzed eHealth use by patients with type 1 and type 2 diabetes mellitus and found a positive correlation between education level and search engine use [8]. These findings underscore that health education and interventions to promote HIT use in adults with diabetes must account for sociodemographic factors. Older adults with diabetes are more often unfamiliar with health technology and potentially adverse to HIT used in telemedicine and eHealth services [18]. Efforts to help older adults and ethnic or racial minorities improve their abilities to navigate and use the internet, and eHealth services may increase HIT use.

Using culturally tailored interventions to better prevent and manage diabetes among minority and underserved populations should be encouraged [1]. In addition, providing guidance on recognizing reliable web-based sources will likely increase their confidence in HIT use.

Moreover, peer-to-peer interactions and increased social media use are growing parts of daily life. Some evidence suggests that implementing social media and increasing the formation of web-based health communities that focus on healthy lifestyle practices may be effective methods to increase confidence in HIT and its use in people with diabetes [19]. Social media interventions have also been associated with hemoglobin A_1c_ reduction and an improved sense of diabetes awareness and empowerment [20]. However, although many patients may have access to the internet, they may not choose to use HIT resources. Positive patient engagement through a networking forum that disseminates reliable health information may help improve patient satisfaction with HIT and increase overall use.

### Associations With Insulin Use and Obesity Status

A higher proportion of eHealth service use was found in patients receiving insulin treatment. Patients with diabetes receiving insulin treatment are likely to require more frequent clinical visits with endocrinologists in addition to primary care physicians and are likely to require more prescription refills for diabetes management. As such, the use of eHealth services may be higher in this subgroup because eHealth services facilitate patient accessibility and convenience of treatment.

Our study found slightly higher HIT use in adults with diabetes and obesity. These findings are in accordance with an earlier survey conducted in Chicago Southside 2012-2013, where Gopalan et al [21] reported that people with measured obesity were more likely to report both general and health-specific HIT use compared with adults with normal weight. Obesity has been associated with difficulties in mobility and other physical activities that may lead to a greater use of HIT. However, the cross-sectional design cannot elucidate the temporal relationship, so we cannot rule out that a greater use of HIT results in a further increase in BMI.

### Limitations

Our study had some limitations. First, the NHIS data were self-reported. Nondifferential misclassifications of BMI categories and HIT use may underestimate the true associations. Second, the NHIS is a cross-sectional survey. It is not possible to draw conclusions about probable causal pathways between sociodemographic factors and HIT use with this study design. Third, as there are other behavior factors associated with HIT use that were not accounted for, it is possible that the significant associations observed in the study were due to unadjusted residual confounders. Fourth, the types of diabetes were not assessed consistently across all survey years; we were not able to fully distinguish patients with type 1 diabetes from those with type 2 diabetes. However, as 90%-95% of adults with diabetes are type 2, we are confident that our results most likely reflect the characteristics of patients with type 2 diabetes [10]. Nonetheless, our study used a large sample size and a national representative sample of the US population with an annual response rate of approximately 70%.

Finally, the Centers for Medicare and Medicaid Services EHR Incentive Program, also known as Meaningful Use, provided incentives to eligible physicians to accelerate the adoption of EHRs [22]. The program began in 2011 and evolved over 3 stages. Although our data overlapped with the Meaningful Use timeline, the NHIS survey questions reflected general adult patients’ behaviors toward the use of eHealth services, not physicians’ responsiveness. Our study was not able to infer the impact of Meaningful Use on HIT in the general population.

### Goals for Healthy People 2030

Newly published goals for Healthy People 2030 have since modified the critical objectives of HIT use, the most relevant of which is to “increase proportion of adults who use information technology to track health care data or communicate with providers” (Health Communication and HIT Objective-7) to a target goal of 87.3% [5]. These goals demonstrate significant increases from the previous 2020 goals. Although these goals reflect the overall desire to increase technology use by the general population, it remains to be seen if individuals with diabetes will meet these standards. In the meantime, more research must be conducted on HIT use and access in people with diabetes to assist this population in achieving these national goals.

### Conclusions

In conclusion, our study found that HIT use in adults with diabetes was slightly lower than the target goals of Healthy People 2020. HIT use differed by several sociodemographic factors. Implementing educational strategies and improving widespread technological accessibility can help ease the transition to HIT and reduce disparities among people with diabetes. It is anticipated that using HIT tools will effectively improve health care quality and increase health delivery efficiency, but further research is needed to delineate the degree of these health benefits translated from the trend of increasing HIT use and the time frame needed for this translation to happen.

## Abbreviations

EHR: electronic health record
HINTS: Health Information National Trends Survey
HIT: health information technology
NHIS: National Health Interview Survey
PR: prevalence ratio
